# Time spent on health related activity by older Australians with diabetes

**DOI:** 10.1186/2251-6581-12-33

**Published:** 2013-07-01

**Authors:** Laurann E Yen, Ian S McRae, Tanisha Jowsey, Nasser Bagheri

**Affiliations:** 1 Australian Primary Health Care Research Institute, Australian National University, Ian Potter House, Corner of Marcus Clarke and Gordon Street, Canberra, ACT 0200, Australia

**Keywords:** Self-management, Time, Health Related Activity, Diabetes

## Abstract

**Aims:**

There is little information available about what people do to look after their health, or how long people spend on health activities. This study identifies key health related activities and time taken as part of self management by people with diabetes. Management planning often lacks information that this study provides that would help clinicians and patients to create manageable and do-able plans that patients can follow.

**Methods:**

Data were collected in 2010 using a national survey of people aged 50 years the National Diabetes Services Scheme. Respondents provided recall data on time used for personal health care, non-clinical health activity; and health service interactions. Data were analysed using Stata 12 and SPSS 19.

**Results:**

While most people with diabetes spend on average less than 30 minutes a day on health-related activities (excluding exercise), the highest decile of respondents averaged over 100 minutes. Time spent increased with the number of co-existent conditions. Taking medication and sitting in waiting rooms were the most frequently reported activities. The greatest amount of time was spent on daily personal health care activities.

**Conclusion:**

The time demands of diabetes for older people can be substantial. Better patient engagement in self management might result from a better match in care planning between the illness demands and the patient time availability, with potential to reduce admissions for hospital care.

## Time spent on health related activity by older Australians with diabetes

Diabetes is a condition in which the patient has a significant role in management; in managing diet and exercise, monitoring and the administration of medicines. As the rate of Type 2 diabetes has increased, and with it, the recognition of the importance of lifestyle factors in good management and outcomes, self-management has become one of the main planks in the patient-doctor partnership for managing diabetes [1]. From a patient perspective, self-management is a mixed blessing: on the one hand, the ability to develop skills and feel in control can be empowering; on the other, the sense of constant vigilance and not performing as well as expected can be de-motivating and distressing [2].

As the website for the Victorian Branch of Diabetes Australia says, “It takes time, energy and commitment to manage your diabetes” [3]. One aspect of diabetes management that has received little research attention is the actual time and task burden on people with diabetes and their family carers in carrying out the array of activities required for management.

In a scoping literature review, Jowsey, Yen and Mathews in 2012 [4] found that while time use surveys in many countries include health questions, these are limited in scope, and are most often reported as aggregates, thus not providing a detailed understanding of either the range of tasks carried out, or the time taken for each of them for people with different conditions. No consensus emerged from the review about a set of specific tasks that could be categorised as self-management, but from that review, these tasks could be broadly categorised as personal self-care, which would consist of activities such as managing medications, monitoring, and treating; non-clinical care, which might include shopping specific to health needs, health education, attendance at support groups; and clinical care, or interactions with professionals and others in the health system.

Russell and colleagues, using data from the American Time Use Surveys (ATUS), have added most to the published knowledge about time use on health activity [5-7]. Jonas et al. (2011) [8] used results from the same survey about health activities undertaken in the previous 24 hours. They analysed information about self-care by adults (using the following definition of self-care: “the personal and medical care performed by the patient, usually in collaboration with and after instruction by a health care professional”, taken from Mosby’s Medical Dictionary). Their study found that of those who reported spending time on self-care, mean time spent on that day was 90 minutes, with 20.6% reporting 2 hours or more. Older people, and those in poorer health were more likely to spend time on self-care than younger people, or people reporting good health. People in poor health, or who were disabled but not in the workforce, reported the highest time use of 3.6 hours and 3.2 hours per week respectively.

In an earlier study, Russell et al. (2008) [7] reported the time costs to patients and carers attending health appointments. Of those adults reporting time on this activity, mean time spent on the survey day on travel was 35 minutes, on waiting was 42 minutes and 74 minutes spent on receiving the service. They also found that almost half of those 65 years or older were accompanied by another adult, thus doubling the time cost to the household.

With specific reference to people with diabetes, Russell et al. (2005) [5] reported the findings from a convenience group of diabetes educators who estimated the time required to carry out activities associated with diabetes that were additional to usual activities. These consisted of taking medications, problem solving, vigilant shopping (checking labels), scheduling appointments, attending support groups and exercise. The consensus of the participants was that experienced diabetics should spend about two hours a day to carry out these tasks; and newly diagnosed people, or those with more complicated health needs, would spend longer.

Understanding both the quantum and type of health related activities in the context of one’s life is important for shaping effective health care. Jeon et al. (2010) [9] report that people managing chronic conditions, both patients and family carers, feel that they are constantly juggling the demands of the condition with other family, social and personal demands. However, the scale of those demands is not included into an analysis of health care costs, leading as some authors propose (see for example Russell (2009) [10]), to a significant underestimate of the true costs of health care. More importantly from the patient perspective, those demands are not included in the planning and scheduling of health services.

The work of May, Montori, Mair and colleagues [11] in developing the concept of the treatment burden carried by people with chronic illness shows this well. Starting from the premise that treatment should be ‘minimally disruptive’ for people with chronic, complex co-morbid illness, the group has developed ideas to enable clinicians and providers to recognise how quickly, and with what impact, the demands of treatment can become so burdensome that the patient simply gives up their role as a collaborator and partner in their health care. Recently, Shippee et al. (2012) [12] have developed a model of cumulative complexity, positing the need for a balance between the workload of chronic illness and the capacity of the patient to fulfil it.

One element of being able to arrive at a health activity workload that is manageable for the patient and their carers, while being affordable to the community, should be some assessment of the tasks people carry out as part of their health care, and how much time this takes.

This paper presents the results of an Australian survey in which we establish a range of health related activities engaged in by Australians aged 50 or more years with diabetes, and how much time they report spending on these activities.

## Method

As part of the Serious and Continuing Illness Policy and Practice Study (SCIPPS), a survey titled “How much work is involved in looking after your health?” was designed to seek information from respondents in four main areas: demographics, health and health services use, time spent by individuals on health related activities and time spent similarly by carers. The survey was tested twice, first by 18 members of a local health care consumer group, and, following modifications, by 29 respondents to a previous survey who had indicated their willingness to assist in future research. Full details of the survey development and conduct have been described elsewhere (Yen et al. (2013) [13]).

In summary, survey forms were posted to a sample of members of three national Australian organisations; National Seniors Australia (NSA), The National Diabetes Services Scheme (NDSS) and Lung Foundation Australia. Since the Lung Foundation Australia group is not included in this report, no further data are given about that sample.

National Seniors Australia is a not for profit member organisation for any Australian aged 50 and over. It has over 285,000 members and provides a range of member services, such insurance brokerage and discounts, as well as advocating on behalf of seniors in matters of government policy. The NSA can be considered broadly representative of older Australians (see McRae et al. (2012) [14]) The NDSS provides a range of subsidised diabetes related products to its 280,000 registered members on behalf of the Australian government, as well as providing information and support services.

A sample of 2,500 registrants was drawn from the NDSS register to obtain a group of respondents all with diabetes (a prerequisite for inclusion on the register). The sample was comprised only of those aged 50 years or more, and was stratified by state of residence, age and gender. A sample of 5000 members of the NSA was drawn, again, stratified by state and age, with an oversampling of older members. Recipients were invited to take part in the survey by completing and returning the paper form sent to them or completing an on line version. Consent to take part was indicated by return of the completed form.

Ethics approval for the study was obtained from the Australian National University Human Research Ethics Committee (Protocol number: 2010/468).

## Data collection and analysis

Respondents were asked specifically how much time, in hours and minutes, they spent on daily personal care, non-clinical health related activity, and health services related activity. This is reported in the paper in terms of minutes per day for daily personal care and hours per month for the other activities undertaken less frequently. Daily activity totals are also estimated with and without exercise, as it is not possible to separate exercise undertaken for health purposes and that for social purposes, and the exercise times tend to dominate other activities.

Data were entered into SPSS files for analysis, with online responses merged electronically into the files. Data are reported as medians and 90th percentiles as the distributions are highly skewed, with confidence intervals derived by bootstrapping. Median and 90th percentile times are reported across the whole population for some activities (generally where most of the population undertake that activity) and for only those who participate in the activity in other contexts. Analysis was undertaken using SPSS Version 19 (Somers, NY, USA) and Stata 12 (College Station, TX, USA).

## Results

The response rate for the Diabetes group was 16.8%; and for the NSA group, 28.4%. While these rates are low, the response group characteristics are in line with the known characteristics of the populations from which they were drawn. Table 1 shows demographic characteristics of the sample, with the NSA sample provided for comparison. As stated above, the NSA sample is broadly representative of the Australian population aged over 50 years. The table shows that males are over-represented in the Diabetes group, as are older participants (aged 70 years and over). Fewer participants in the Diabetes group are in the workforce, an effect which holds true within each age group. Despite diabetes being a requirement for membership of the NDSS, Some 3% of the sample did not report having a diagnosis of diabetes (Table 2).

**Table 1:** Shows the demographic characteristics of the Diabetes sample group, with the NSA sub-sample used as a comparison

	**Diabetes sub-sample**	**NSA sub-sample**
	**(N = 427)**	**(N = 1,432)**
*Percentage of subsample population*		
*Gender*		
Male	56.5	39.9
Female	43.5	60.1
*Age*		
Less than 60 years	24.9	26.8
60-69 years	35.4	49.2
70-79 years	26.6	16.9
80 years and over	13.1	7.1
*Region of residence*		
Major City	56.7	57.6
Inner Regional	29.9	27.9
Outer Regional	12.0	11.3
Remote and very remote	1.4	3.2
*Employment*		
Working	22.8	36.7
Not working	77.2	63.3
*Self Assessed Health*		
Excellent, very good or good	61.4	83.2
Poor or fair	38.6	16.8

**Table 2:** Shows the percentage of the diabetes sample who carried out each of the listed activities, and for those who did engage in them, the time spent at the median and 90th percentile

**Activity**	**Proportion reporting this activity**	**Median time if undertaking this activity**	**90th percentile of time if undertaking this activity**
***Health related activity***	**% active**	**Median**	**90th percentile**
	**N = 427**	**(95% CI)**	**(95% CI)**
***Personal care activity***			
On most days how much time do you spend…			
		*Minutes per day*	
1. Sorting your medication?	65.0	5 (5–5)	15 (9.5-20.5)
2. Preparing your medication?	52.5	5 (4.0-6.0)	15 (8.6-21.4)
3. Taking your medication?	82.4	2 (1.1-2.9)	10 (7.9-12.1)
4. Carrying out treatments?	21.3	5 (1.9-8.1)	30 (17.8-42.2)
5. Testing or monitoring health?	68.1	5 (5–5)	15 (12.0-18.0)
6. Preparing special foods?	17.9	30 (30–30.0)	60 (15.0-105.0)
7. Taking exercise/stretching?	61.6	30 (30–30)	90 (61.5-119.5)
Total Personal Care Activity excluding exercise	91.2	15 (12.6-17.4)	70 (67.1-92.9)
Total Personal Care Activity including exercise	93.2	41 (35.3-46.7)	142 (113.2-170.8)
***Non-clinical health activity***			
In the last month how much time did you spend…			
		*Hours per month*	
1. Shopping for medicine, equipment or disposables, other necessary health items for yourself?	71.9	0.5 (0.4-0.6)	2 (1.3-2.7)
2. Shopping for special foods you may need for yourself?	27.6	1 (0.7-1.3)	4 (2.8-5.2)
3. Attending rehabilitation programs?	3.7	2 (1.0-3.0)	6 (0.3-11.7)
4. Attending health education or self-management programs?	8.0	1 (.02-1.8)	4 (0—16.5)
5. Attending support groups, such as cancer or diabetes groups?	6.9	1 (1.0-1.9)	4 (0–16.8)
6. Looking for and reading health information?	30.1	1 (0.6-1.4)	4 (0–8.7)
Total Non Clinical Health Activity	80.8	1 (0.8-1.2)	6 (4.1-7.9)
***Health service related activity***			
In the last month how much time did you spend…			
		*Hours per month*	
1. Organising appointments for yourself?	58.9	0.2 (0.2-0.2)	1 (0.8—1.2)
2. Organising travel to and from health-related appointments?	26.3	0.5 (0.4-0.6)	2 (0.9-3.1)
3. Travelling to and from health-related appointments, including support groups?	53.8	0.8 (0.5-1.1)	6 (3.4-8.6)
4. Sitting in waiting rooms?	74.5	0.5 (.03-.07)	3.2 (2.1-4.2)
5. With the doctor or health professional for consultation, advice or treatment?	62.5	0.5 (0.5-0.5)	3 (1.6-4.4)
6. Having blood tests, X-Rays or other tests?	55.4	0.5 (0.3-0.7)	2 (1.5-2.5)
7. Having other medical treatments?	3.7	1 (0–3.2)	6 (0–12.4)
Total Health Service Related Activity	82.4	2.2 (1.8-2.6)	13 (10.1-15.9)
*Hours per month*			
Total Reported Time excluding exercise	95.1	12.3 (10.2-14.3)	51.5 (42.6-60.4)
Total Reported Time including exercise	95.5	26.2 (22.6-29.8)	91 (76.4-105.6)

The continuing conditions most reported in addition to diabetes were hypertension (60.8%), arthritis (34.2%), and heart disease (23.9%). Almost a quarter of the respondents reported having been diagnosed with cancer at some time. A quarter reported being diagnosed with chronic pain; and 20.4% reported having been diagnosed with depression.

As would be expected those with diabetes are in poorer health than those in the wider community, with 38.6% of the diabetes sample reported being in poor or fair health compared to 16.8% in the NSA sample.

Over 90% of respondents reported spending time most days on personal health care activities; with taking medication as the most frequently reported activity. Over 80% reported spending time over the last month in non-clinical health related activity, most frequently on shopping for health items. A similar percentage reported time spent on interactions with health services.

The median total time spent each month was, however, generally quite small, with 7.5 hours spent on personal health care, rising to 20.5 hours when exercise is included; 1 hour a month on non-clinical health related activity; and 2.2 (2 hours 12 minutes) hours on activities related to health services interactions. The total time daily is on average just less than 25 minutes; and just under an hour a day if exercise is included.

However, those at the 90th percentile reported significantly greater amounts of time spent on health related activity. Most commonly, those at the 90th percentile reported times three or four times greater than those at the median, but up to 5–7 times greater for taking medication, carrying out treatments, and travelling to appointments. The following sections provide greater detail on specific interactions.

## Managing medicines

Most respondents (82.4%) spent time taking medications. Almost two thirds reported time spent sorting medication and about half said they spent time most days preparing medication. The median time spent doing this over a month, for all three activities, was around 6 hours, or 12 minutes a day.

Within the sample, 406 respondents reported the number of prescribed medicines used. Time spent on each of the three medicines related activities, sorting, preparing and taking medicines, increased directly with increasing numbers of medicines. While the time taken was generally small (medians of 5 minutes or less for each activity) those at the 90th% taking 10 or more medicines each day reported spending 25–30 minutes on each of the activities (Table 3).

**Table 3:** Number of medicines X time taken for each medicine activity (minutes per day)

** Activity**	**N**	**Proportion undertaking this activity**	**Median time if undertaking this activity**	**90th percentile of time if undertaking this activity**
*** Number of medications***		**% active**	**Median**	**90th percentile**
			**(95% CI)**	**(95% CI)**
			***Minutes per day***	
Activity: sorting your medication?				
1-2 medications	44	44.5	2 (0.4-3.6)	10 (0–27.9)
3-5 medications	188	70.6	3 (1.0-5.0)	15 (9.6-20.4)
6-10 medications	143	69.4	5 (1.7-8.3)	15 (1.4-28.6)
>10 medications	31	71.2	10 (5.9-14.1)	30 (19.7-40.3)
**Total**	**406**	**67.3**	**5 (5–5)**	**15 (9.8-20.2)**
Activity: preparing your medication?				
1-2 medications	44	37.7	1 (0.3-1.7)	10 (0–28.0)
3-5 medications	188	47.8	2 (0.4-3.6)	10 (5.2-14.8)
6-10 medications	143	63.7	5 (5–5)	20 (10.8-29.2)
>10 medications	31	70.4	5 (0–10.8)	30 (18.6-41.4)
**Total**	**406**	**53.8**	**5 (4.2-5.8)**	**15 (8.6-21.4)**
Activity: taking your medication?				
1-2 medications	44	72.7	1 (0.5-1.5)	5 (4.2-5.8)
3-5 medications	188	86.2	2 (1.7-2.3)	10 (6.1-13.9)
6-10 medications	143	86.9	3 (1.0-5.0)	10 (5.1-14.9)
>10 medications	31	90.5	5 (3.3-6.7)	30 (0–69.5)
**Total**	**406**	**85.3**	**2 (1.2-2.8)**	**10 (8.1-11.9)**

## Testing and monitoring

People with diabetes are recommended to undertake a number of self-monitoring activities, as outlined in the introduction. More than two thirds of the group reported undertaking testing or monitoring activities daily. Median time expenditure each day reported was 5 minutes and the 90th percentile 15 minutes. There was no clear pattern with age.

## Time spent on non-clinical health related activity

Shopping for health items was the most reported activity at almost 72% of the sample with 28% reporting time spent on shopping for special foods. Only a small percentage of the sample had spent time in the last month attending health education programs (8.0%) or support groups (6.9%). This contrasted with the 30.1% of participants who reported spending time reading health information, with a median time spent of an hour a month.

## Time spent on health services related activity

The service related activity reported by the highest percentage of respondents was spending time sitting in waiting rooms (74.5%), with a median time for this activity over the past month of 30 minutes. This was higher than the percentage of people reporting spending time in consultation with a doctor or other health professional (62.5%). Just over 55% of respondents had spent time in the previous month having ‘blood tests, X-rays etc’, which may explain some of the gap between those waiting for, and those seeing, their doctor or other health professional.

## Does the number of chronic conditions make a difference to time spent?

The time spent on the three medicine related activities generally increased with the number of chronic conditions reported. Again, median times for each activity were small at between two and five minutes on each. For those at the 90th percentile, time increased broadly in step with the number of chronic conditions to a maximum for participants with five or more conditions of 20 minutes per day for sorting medications, 30 minutes for preparing medications and 10 minutes for taking medications.

## Self-rated health and time spent

Time spent on each activity set increased as self-rated health declined, as shown in Tables 4, 5, 6 and 7 with those with poorer health generally showing higher time use while not all differences are statistically significant. The two exceptions are the median personal activity time including exercise, where it could be hypothesised that those in poor health are less able to exercise, and the 90th percentile on health services activity where confidence intervals are very wide.

**Table 4:** Time spent on personal care excluding exercise and self-reported health

**Self-reported health**	**N**	**Percent with personal care time greater than zero**	**Personal care activity excluding exercise (minutes per day)**	
			**Median and percentile include zero values**	
		**%**	**Median (95% CI)**	**90th percentile (95% CI)**
Excellent/ very good	89	87.8	6 (3.7-8.3)	35 (12.6-57.4)
Good	172	93.4	15 (10.6-19.4)	81 (60.9-101.1
Poor/Fair	161	91.1	18 (12.9-23.0)	85 (64.9-105.1)
**Total**	**422**	**91.3**	**12 (9.0-15.0)**	**70 (56.4-83.6)**

**Table 5:** Time spent on personal care including exercise and self-reported health

**Self-reported health**	**N**	**Percent with personal care time greater than zero**	**Personal care activity including exercise (minutes per day)**	
			**Median and percentile include zero values**	
		**%**	**Median (95% CI)**	**90th percentile (95% CI)**
Excellent/ very good	89	92.2	30 (20.9-39.1)	95 (59.3-130.7)
Good	172	94.6	50 (39.9-60.1)	150 (117.0-190.0)
Poor/Fair	161	92.8	37 (28.6-45.4)	165 (100.8-229.2)
**Total**	**422**	**93.3**	**38 (32.0-43.9)**	**130 (104.5-155.4)**

**Table 6:** Time spent on non-clinical activity and self-reported health

**Self-reported health**	**N**	**Percent with non-clinical time greater than zero**	**Non-clinical but health related (hours per month)**	
			**Median and percentile include zero values**	
		**%**	**Median (95% CI)**	**90th percentile**
Excellent/ very good	89	75.5	0.33 (0.16-0.51)	6 (1.5-10.5)
Good	172	87.1	0.67 (0.58-0.76)	4.5 (1.7-7.3)
Poor/Fair	161	78.0	1.0 (0.74-1.26)	6 (3.4-8.6)
**Total**	**422**	**81.0**	**0.5 (0.41-0.59)**	**5.25 (3.6-6.9)**

**Table 7:** Time spent on health services related activity and self reported health

**Self-reported health**	**N**	**Percent with health services related time greater than zero**	**Health services related activity (hours per month)**	
			**Median and percentile include zero values**	
		**%**	**Median (95% CI)**	**90th percentile**
Excellent/ very good	89	76.1	1.0 (0.70-1.30)	10.7 (4.0-17.4)
Good	172	85.1	1.58 (1.24-1.93)	8.7 (5.8-11.5)
Poor/Fair	161	83.3	2.50 (1.15-3.85)	14.7 (10.7-18.6)
**Total**	**422**	**82.4**	**1.67 (1.4-1.9)**	**11.6 (8.9-14.3)**

## Does where the person lives have an effect?

Of the 416 participants in the sample who reported their area of residence, 218 (52.4%) lived in a state capital city. The remaining 198 participants lived in regional or rural areas.

There was little difference between city and country dwellers in the time spent overall on daily personal care or non-clinic health care. However, people living in major cities spent more time on “shopping for health items” and on “shopping for special foods” (medians 0.75 and 0.5 of an hour per month; 90th percentiles 1.5 and 1.0 hours per month).

The total time spent on health service related activity was higher for those living in major cities than in small cities, or rural and remote regions (median city 1.71 hours per month: non-city 1.42 hours per month). However, this was reversed for travel time alone, where those living in major cities spent a mean time of 1.37 hours a month on health related travel, compared to 1.72 hours spent by those living in rural and regional areas. At the 90th percentile, travel time for rural and regional participants was double that for those living in major cities (2 hours per month compared to 4 hours per month).

In summary, major city living participants spent more time than country participants shopping, and accessing health services; but regional and rural people spent more time travelling to services.

## Does the number of health professionals consulted have an effect?

Participants were asked to identify which types of health professionals (such as GP, Specialist, Allied health) they had consulted in the previous three months, and how often. Time spent increased with the number of professionals seen, as shown in Table 8 below, and the differences between each level of use were significant (except zero to 1–2 where the zero group is based on a very small sample).

**Table 8:** Time spent and number of professionals consulted

**Number of health professionals Seen**	**N**	**Percent with health services related time greater than zero**	**Health services related activity (hrs/month)**	
			**Median and percentile include zero values**	
		**%**	**Median (95% CI)**	**90th percentile**
Zero	45	61.1	0.67 (0–1.36)	11.3 (2.3-20.4)
1-2	102	74.1	0.83 (0.52-1.15)	2.5 (0–5.2)
3-5	159	88.7	2 (1.58-2.41)	9 (6.3-11.7)
>5	121	88.7	4 (2.64-5.36)	17.7 (7.4-27.9)
**Total**	**427**	**82.4**	**1.67 (1.40-1.93)**	**11.6 (8.9-14.3)**

Participants were asked whether or not they had been given a management plan by their GP or other health professional, with 44% of the group reporting that they had and 48% reporting they had not. Having a management plan was linked to *higher* time expenditure on each of the three categories, adding 50% to the personal care activity time and 33% to non-clinical health activity time but only 10% to the time spent on health service related activity.

## Discussion

In summary, this study describes the complex picture of chronic illness in this group of people aged 50 years and more and living with diabetes in Australia. Since the recommendations for self-care for diabetes are international, we believe that these results are of interest to the broader community of researchers and practitioners in the field of diabetes.

Almost a quarter of the group reported having 5 or more conditions. A similar number reported consulting more than 5 different professionals in the previous three months. Perhaps the most startling finding here is that 44% of respondents reported taking 6 or more *different* prescription medicines daily. Increasing numbers of conditions, medications and health professionals consulted were each associated with increasing time spent on health related activity as was poorer self-reported health. While each of these findings alone may be accounted for in part by factors associated with ageing, their value is in pointing to the increasing complexity of health activity that occurs at a time when capacity to manage is likely to be decreasing.

These findings give some indication of the complex management required by patients and by their treating teams. Management comes at a cost, and the finding that respondents with a management plan reported greater time spent on health related activity suggests that the treatment burden, as explored by May et al. [11] is measurable in time terms, as well as in complexity terms. The findings cannot show whether those whose health needs are more complex and so possibly more time consuming are those who have a management plan, but since over 40% of respondents said they had a plan, this is likely to include people with a range of severity. However, it is highly relevant to the discussion in Shippee et al. (2012) [12] in which the authors argue the need to balance the complexity of the patient task with capacity of the patient to undertake it so that, inter alia, patients remain engaged in their care. These findings provide information about one of the key and practical capacity issues.

The findings also identify particular activities that people carry out as part of looking after their health. Most of this group spent time on aspects of medication management, and on testing and monitoring. Fewer than 10% of respondents reported spending time on health education or support groups (although this is quite time demanding for those who did attend). A majority of respondents reported spending time on health service interactions, with the most reported activity being, rather dismally, sitting in waiting rooms. While the set of activities we have included is far from exhaustive, and is focused on practical and logistical activity as opposed to the emotional and spiritual activity that also accompanies chronic illness, it nonetheless provides the first information available on time spent on these tasks, and the likely pattern of self-care that people undertake.

The actual time taken by this group in looking after their health is generally much less than the times estimated by, for example, Russell in her convenience sample of diabetes educators where 2 hours was judged to be the time required each day. The greatest amount of time in our sample was spent on daily personal care activities but this was generally less than 30 minutes a day, with an additional 30 minutes for exercise. However, for those in the top decile of time use, total time was almost two hours per day without exercise and three hours per day with exercise. This suggests either that the time estimates given to Russell by diabetes educators relate to the upper end of time spent, or that people with diabetes are routinely spending less time on self-management than professionals recommend. In either case, this study suggests further research would be useful in determining where patient reality and professional reality differ.

## Limitations

This study limited reporting to a series of health related activities, and those mostly practical and logistical activities. As a result, the study does not report the time spent thinking about, being worried by, or planning relating to managing diabetes, nor time required to rest.

The low response rate to the questionnaire may have skewed the respondent group, although we are not able to say in what way. The sample pool is seen to be strongly representative of Australians with diabetes, since is it estimated that it includes between 80 and 90% of all people diagnosed with diabetes in Australia [15]. Those not included may be those who manage their diabetes without medication or need for testing; or who choose to buy non-subsidised diabetes products. We noted that a small number of respondents did not identify themselves as having diabetes, despite this being a requirement for membership of the NDSS, which we believe may reflect different ‘names’ used by both doctors and patients for the condition.

The study asked which activities were undertaken, but not how often. This information would have enabled a more fine-grained analysis of some activities, particularly those relating to health services use.

As the study is a recall study of respondents’ understandings of their time use it will suffer from various forms of recall bias. However, this approach was taken in preference to a diary based approach as it enabled the questionnaire to cover a much a longer period and hence more events than would be possible with a diary.

## What do these findings add to our understanding of the management of diabetes?

Most of these respondents had multiple chronic conditions, in addition to any less enduring conditions they faced. They took multiple medications, and saw multiple health professionals and with poorer health, treatment burden increased as did the demand on personal time. Those at the highest decile of time use spent 2 to 3 hours a day (depending on whether exercise was included in the figuring) on managing their health.

This is strong support for the need to address diabetes as part of an overall health plan; and to recognise the potential hazards attached to the complexity of holistic management in a system that largely continues to manage condition by condition. In an earlier study in this series, Yen et al. (2011) [16] found that patients with chronic illness were frustrated by the failure they saw amongst their treating professionals to communicate and coordinate care, and these results strongly support the need for a co-ordinating function to assist patients in negotiating their care demands. In a small Australian study of information continuity to support coordination, Banfield et al. (2013) [17] found that information useful to patient coordination rarely travelled between services, or between different elements within a service, leading to a concern that the activities contained in management plans may not be themselves well integrated and coordinated. Our finding that having a management plan is associated with greater time spent needs further research to establish the nature of the relationship.

## Conclusion

People with diabetes carry out a range of practical tasks associated with their health care, all of which need to be factored in to their family and other commitments. While most people report spending less than an hour a day on these activities in total, those who have complex management, through having multiple conditions, multiple medications and multiple health professionals are high time users. For those the top 10% of time users, the time spent each month can exceed 50 hours even without accommodating exercise time.

The impact of complex treatment demands, such as multiple medications and professional interactions is amenable to system intervention- such demands could be made more manageable and streamlined by more thoughtful coordination and better continuity.

## Competing interests

All authors declare that they have no competing interests.

## Authors’ contributions

LY led the conception, design and conduct of the study, contributed to the analysis of the results and wrote the paper; IMcR contributed to the conception and design of the study, led the analysis of the results and contributed to the writing and revisions of the paper; TJ contributed to the conception and design of the study and to the writing of the paper; NB contributed to the analysis of the results and contributed to the revision of the paper. All authors read and approved the final manuscript.
